# Driving and Restraining Forces in the Implementation of Information Systems in the Public Sector: Scoping Review

**DOI:** 10.2196/71575

**Published:** 2025-06-11

**Authors:** Arja Lemmettylä, Ulla-Mari Kinnunen

**Affiliations:** 1 Department of Health and Social Management, Faculty of Social Sciences and Business Studies, University of Eastern Finland, Yliopistonranta 1, Kuopio, 70210, Finland, 358 443382518; 2 Research Centre for Nursing Science and Social and Health Management, Kuopio University Hospital, Wellbeing Services County of North Savo, Kuopio, Finland

**Keywords:** information systems, implementation, public sector, digital transformation

## Abstract

**Background:**

Public sector organizations increasingly adopt information systems (ISs) to improve economic efficiency, service quality and overall adaptability. These projects represent substantial financial investments, making their success critical for organizational performance and societal impact.

**Objective:**

This scoping review aimed to identify the driving and restraining forces influencing IS implementation in public sector organizations and to explore strategies that support successful change processes.

**Methods:**

A total of 25 peer-reviewed articles were analyzed using Lewin’s change theory to categorize and interpret driving and restraining forces. In addition, the narrative emerging from previous research on IS implementation was examined to explore how previous research portrays the success of IS implementation processes.

**Results:**

The findings highlight that IS implementation is influenced by 6 domains: organizational practices and challenges, technological factors and barriers, management practices and issues, change project factors and challenges, end user factors and concerns, as well as institutional factors and barriers. Key driving forces include leadership support, stakeholder involvement and system usability, while restraining forces encompass user resistance, technical challenges, and organizational silos.

**Conclusions:**

Despite the challenges, IS implementation offers significant opportunities for improving public sector operations and societal outcomes. Addressing restraining forces and leveraging driving forces is essential for achieving sustainable digital transformation. This study provides actionable insights for future IS implementation in the public sector.

## Introduction

The implementation of information systems (ISs) is one of the greatest transformation challenges that organizations face. Digitalization of services and operations is fundamentally transforming public sector organizations. It is seen as a key component of administrative reforms, and as a solution to high public expenditure, inefficiency, increased user involvement, and the need for greater transparency in public administration [1,2]. In addition, the digital transformation observed in other sectors has led citizens to expect real time, high-value services from the public sector [3]. In this study, public sector organizations are defined as government-funded and operated entities, such as health and social care institutions.

In health care, ISs play a critical role in operational processes, directly influencing health service outcomes. The term IS refer to a set of digital technologies and processes designed to collect, process, store, and disseminate information within organizations, including tools like electronic health records (EHRs) and other information technology (IT) solutions to support organizational operations. The digitalization of health care has potential to enhance care effectiveness, improve cost efficiency, and enable new service delivery methods [4,5].

Despite the advanced quality of health ISs, they also represent significant financial investments [6]. Furthermore, IS development is filled with challenges [7-10]. There are numerous ways to cause large information technology projects to fall short of expectations [11-13]. The failure rates have been persistently high [14] with estimates suggesting that up to 60%‐70% of system projects in health care fail [15,16]. Despite the best intentions in software development, delays, failures, and complete abandonment remain possible outcomes [8,17]. The success of IS implementation refers to their ability to meet organizational needs, achieve user acceptance, and deliver intended outcomes, such as operational efficiency and financial sustainability.

Successful adoption of new technology is supported by considering factors related to the organization, people, work, and technology [18,19]. IS-related changes require effective leadership [20-22] and capability to modify practices both at the individual and organizational levels to achieve success [18,23,24]. However, the importance of context and process is often overlooked [25,26]. Implementation in this context refers to the process of planning, deploying, and integrating ISs into organizational workflows, encompassing technical aspects as well as organizational factors such as stakeholder engagement and change management.

Given the clear necessity for the IS development and increasing financial constraints faced by public sector organizations, successful implementation is essential. The complexity of these projects highlights the need for deeper understanding of the forces that influence their success. Previous research has approached IS implementations from multiple perspectives, including their impact on organizations, services and operations [15,27], individual responses to technology adoption and resistance [16,28-30], and project management and governance [11,31]. However, these studies have often remained fragmented, addressing isolated aspects rather than forming a cohesive understanding of IS implementation processes in public sector organizations. A comprehensive examination from the public sector’s viewpoint provides a novel contribution to the existing literature. The purpose of the study is to improve the understanding of the factors influencing IS implementation in public sector organizations and to provide insights into preparing for a successful change process.

The analysis was conducted using Lewin’s change model and its force fields [32], which categorizes factors as either driving or restraining forces [33-35]. According to model, change is influenced by driving forces which push toward change and support it, and restraining forces which seek to maintain the status quo and resist change [36]. With Lewin’s model, it is possible to analyze and identify forces that either propel the change forward or create obstacles that can halt the desired change altogether [34]. Using Lewin’s model, this study reveals new insights into the forces influencing IS implementations, building on previous research. Both the driving and restraining forces are identified and analyzed, offering a deeper understanding of the dynamics during a system adoption in public sector organizations.

Research questions are: (1) What are the driving and restraining forces in the implementation of ISs within public sector organizations? (2) What kind of narrative emerges about the success of the change process?

## Methods

Research questions were addressed through a scoping review. This method allows to summarize the breadth and depth of the research field on the topic [37]. It helps to identify relevant literature, clarify concepts, and map research activity within the subject area [38]. Through a scoping review, existing knowledge can be identified, evaluated, interpreted, and combined [39,40].

Population, concept, context method [41] was used for topic definition and search strategy formulation. The databases selected were Scopus, Web of Science, and PubMed due to the relevance of the topic and the coverage.

The search queries and results are detailed in Table 1. The database searches were subject to general inclusion criteria described in Textbox 1.

**Table 1.** Research queries and results.

Database	Research queries	Results (n=509), n
Scopus	(TITLE-ABS-KEY (“information system*” OR “health information system*” OR “computer system*“) AND TITLE-ABS-KEY (implementation OR deployment) AND TITLE-ABS-KEY (transformation OR change OR “organizational change”) AND TITLE-ABS-KEY (“public sector” OR “health care organization*” OR “social care organization*"))	22
Web of Science	(((ALL=(“information system*” OR “health information system*” OR “computer system*“)) AND ALL=(implementation OR deployment)) AND ALL=(transformation OR change OR “organizational change”)) AND ALL=(“public sector” OR “health care organization*” OR “social care organization*")	23
PubMed	(((((((“information system”[Title/Abstract] OR “health information system”[Title/Abstract] OR “computer system”[Title/Abstract]) AND “implementation”[Title/Abstract]) OR “deployment”[Title/Abstract]) AND “transformation”[Title/Abstract]) OR “change”[Title/Abstract] OR “organizational change”[Title/Abstract]) AND “public sector”[Title/Abstract]) OR “health care organization”[Title/Abstract] OR “social care organization”[Title/Abstract])	464

Textbox 1. **Inclusion criteria.**

- Available electronically
- Peer-reviewed
- Accessible full text
- Published in English
- Published between 2018‐2023
- Relevant to the research questions

## Results

### Overview

**Figure 1.**
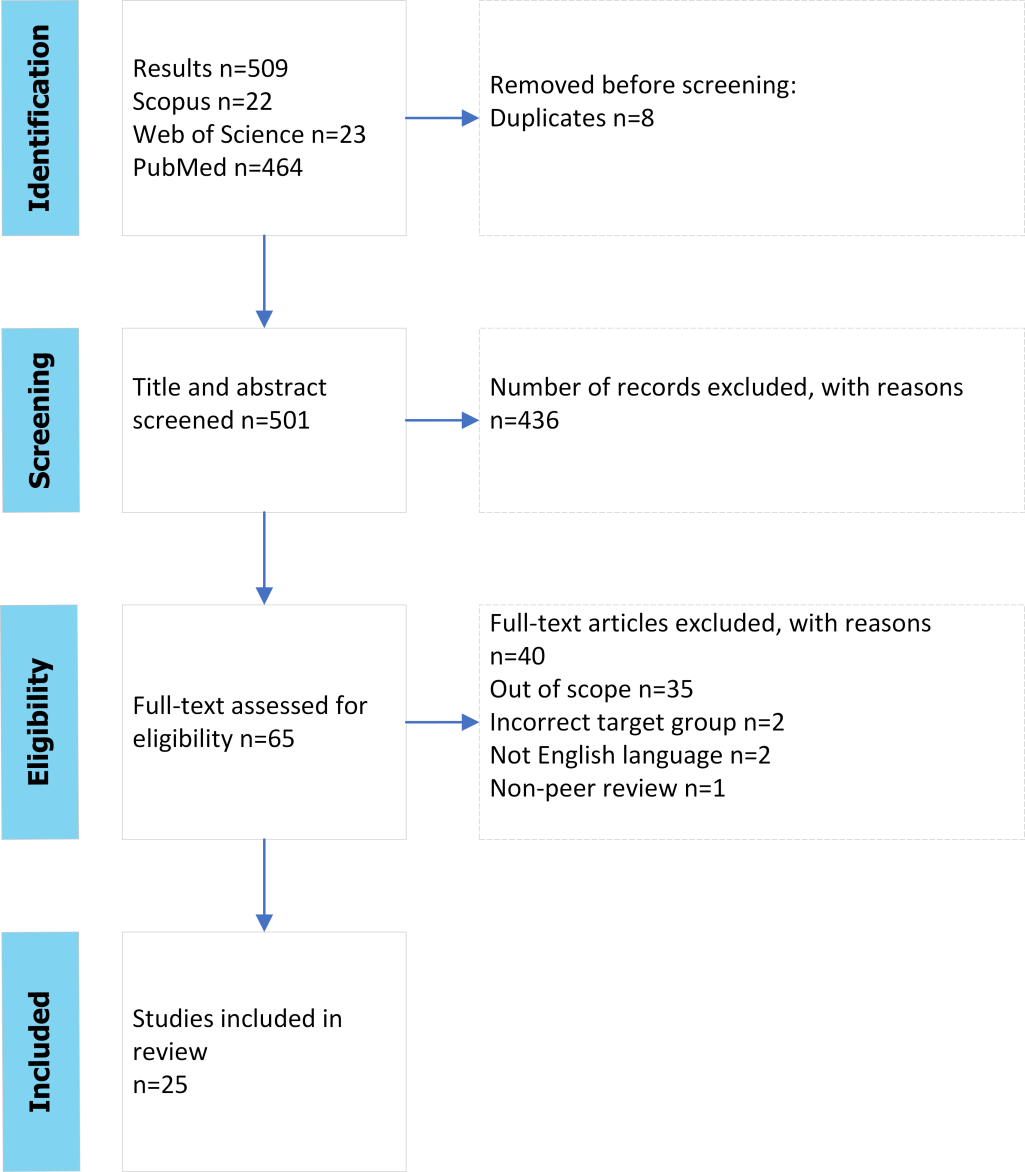
PRISMA (Preferred Reporting Items for Systematic Reviews and Meta-Analyses) flow diagram [42].

The data handling process is illustrated in the PRISMA (Preferred Reporting Items for Systematic reviews and Meta-Analyses) flow diagram (Figure 1) [42] and the PRISMA-ScR (Preferred Reporting Items for Systematic Reviews and Meta-Analyses Extension for Scoping Reviews) checklist is present as Checklist 1. The final study included 25 primary research articles, and the data are summarized in Multimedia Appendix 1. Narratives about IS implementations presented in research articles are summarized in Multimedia Appendix 2. The data analysis was conducted by using force fields of the Lewin’s change model as it provides a framework to assess the success of IS reform in relation to planning, implementation, and user acceptance [34,35].

**Figure 2.**
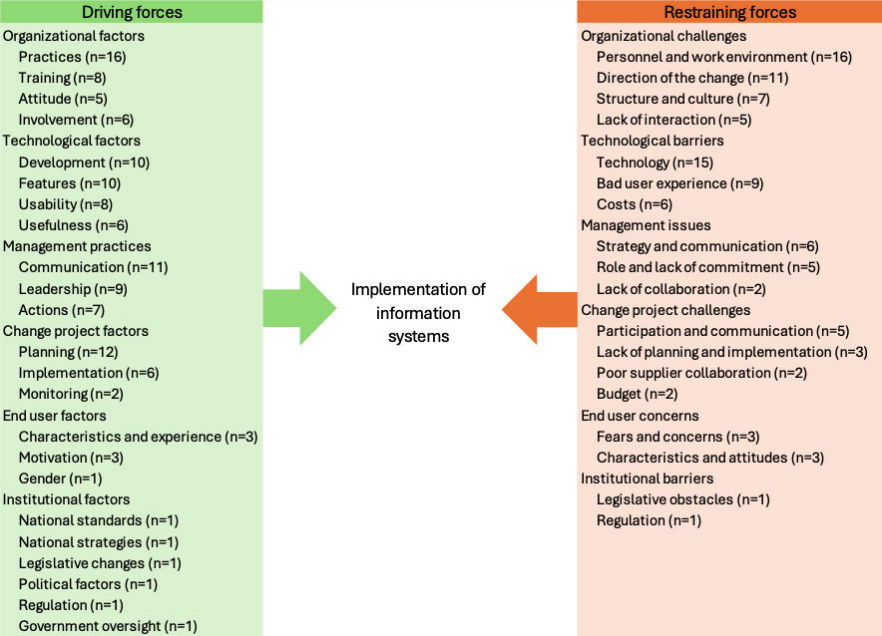
Driving and restraining forces affecting on implementation of information systems.

The articles were published between 2018 and 2024. The publications originated from a total of 15 different countries and 5 different continents. Most of the studies were qualitative (n=18). In addition, there was 1 mixed methods study, 3 quantitative studies, and 3 literature reviews. Public sector domains were health care (n=18), government agencies (n=3), taxation (n=2), and social welfare (n=2). Forces that drive and restrain the implementation of ISs in public sector organizations were grouped into broader categories (Figure 2). Subgroups were created under the categories, highlighting the most frequently mentioned factors identified from the data (Figure 2). While numerous studies addressed avoidable restraining factors, the emphasis in the data was on the driving factors.

### Driving Forces

The implementation of ISs in public sector organizations is influenced by a range of driving forces. However, the majority individual forces fall under the categories of organizational and technological factors.

#### Organizational Factors

Existing practices within the organization can significantly promote the adoption of new systems. Key factors that enhance the personnel’s readiness for change include openness [43], encouragement [44], providing safe space for expressing concerns [44], and proactively addressing issues [45]. If the organization learns through negotiation and interaction [465], it is more capable of implementing changes. Centralized coordination and standardization functions [46], implementation practices [47], and decision-making capability [48] within the organization are also important. Change is facilitated if the organization is built around integrated processes that evolve with changes [49] and if these processes are monitored and adjusted when necessary [50]. IT governance reform [48] should be considered as market-driven solution development [46] emerging as a driving factor. The use of a same brand [51] can promote the implementation of ISs in large entities. In extreme cases, personnel can be mandated to use the system [52].

Training of the personnel and end users was highlighted in 8 studies [44,51-57]. Training should occur before the implementation of the ISs, but postimplementation training and guidance should also be emphasized. In addition to training, participation was noted in 6 studies in the dataset [43,52,55,58-60]. All stakeholders, including frontline professionals [58], should be involved from the very beginning.

Attitudinal factors can manifest within the organization as a whole and among its members. In health care, there is a noticeable sense of moral and ethical obligation among professionals to promote change [59], particularly among those in leadership roles. A positive attitude toward innovation [43] and determined efforts [51] supports the change. The organization’s ability to create space for creativity [43], agility, and communication along with confidence in the success of the change [59] eases the process.

#### Technological Factors

Key aspects of development include good software designing [61] and its feasibility [46], implementation strategy [61], testing and validation practices [55,61], and the sensitivity of software developers to the client’s need [61]. Innovative solutions are more likely to emerge through local development [46], but it is crucial that solutions also pass pre-established quality gates [53].

ISs possess various features that either drive or restrain their implementation. The most significant driving factor is solution that support workflows [50,55,57,62]. Other factors include the system’s observability [54], interoperability [55], and compatibility [54]. The more the system is used, the more likely the usage will increase [56]. The IS must also fit within the organizational culture [53]. When the IS is perceived to provide more relative benefits, it further drives its adoption [54].

Ease of use was identified as a driving factor in 4 studies [50,56,58,62]. Usability of the IS was discussed in 2 studies [47,55]. It is essential that needed information must be accessible [62] and the system is available for user testing [54]. Perceived usefulness [55,56,58] and even forecasts of positive impacts [56] are considered driving factors. It is crucial that the IS has the potential to enhance aspects such as the quality of care [45] and it supports professionals in performing their tasks [63].

#### Management Practices

In the context of implementing ISs in public sector organization, management involves leadership activities, communication skills, continuous assessment, and collaboration. In terms of communication, the involvement of individuals in leadership positions emerged as significant factor [43,59]. At a more detailed level, it is essential for management to convey expectations [50] and objectives [60] related to the change. Ensuring that the organization’s vision is clearly understood in the context of the change is important [64]. Expectations are placed on leaders’ communication, as listening to concerns and doubts [55,60], thereby fostering greater understanding [59]. Managers must be able to address questions regarding changes [59] and be capable of negotiating potential tensions [65]. They should also possess the capability to motivate users [58].

Overall, leadership and change management were highlighted in 3 studies [44,52,55]. Those in leadership positions must ensure the success of the change process [60] and have confidence in the success of the change [65]. The alignment of differing perspectives [64] and the involvement of leaders [48] are also seen as driving factors. Notably, Fennelly et al [55] observed that successful implementation is more likely when the change is not managed directly by top leadership as otherwise the data emphasized the importance of active leadership engagement.

When leading the change, management should focus on preventing the consolidation of the conflicting views [64] and addressing any ambiguities or issues [60] to promote the acceptance of innovations. Managing the change process [58] and monitoring potential performance deficiencies [49] are key factors in implementation process. Management must be able to develop and communicate context-specific goals [58] for the change. Although it was previously noted that change should not be directly led by top management, a collaborative IT group led by management can promote the IS change [49].

#### Change Project Factors

At the beginning, it is essential to secure resources [55] and ensure clear roles and responsibilities [48,55]. A project team must be established [52] and the supporting structures for the change processes addressed [66]. Further driving factors include stakeholder involvement from the perspective of the change project [66] and the early engagement of various stakeholders [45]. Maintaining strong and trustworthy relationships with software vendors and consulting firms is critical [55]. In early stages of the change project, attention should be paid to the implementation strategy [55], as well as to the practices for adoption that will arise in later phases of the process [43].

During the implementation phase, the importance of the project management team and governance [55] becomes important. A well-planned implementation process [55] supports the change management of the project [50]. Establishing a command center for the adoption phase [48], involving operational-level leadership roles [55], facilitates the actual system rollout. The change project must have clear and measurable goals from a monitoring perspective [55]. Strong communication practices are essential to promoting the desired practical implementation [46].

#### End User Factors

Regarding user characteristics, a high level of education [56], technological proficiency [62], and overall competence [53] are identified as factors that drives the adoption of the IS. In addition, the motivation [44], commitment [44], and efforts [65] of end users are critical. In 1 study, being female was also identified as a driving factor [56].

#### Institutional Factors

Institutional factors refer to government guidance and external elements that influence the organizational environment. They are essential factors from the perspective of public sector organizations. National strategies [51] and standards [55] guide the development of the social and health care sectors. Legislative changes [51] and regulations [67] can support or accelerate system reforms within organizations. Government oversight [52] and political factors [67] are also driving forces.

### Restraining Forces

Most individual factors restraining the implementation of ISs fell under the categories of organizational challenges and technological barriers. In total, 4 studies [43,47,51,62] did not address restraining factors.

#### Organizational Challenges

Concerns related to personnel and the work environment include various issues as the loss of professional identity, patient safety, reduced visibility of work and workforce retention, which negatively impact change processes related to ISs [44]. There are concerns about the possibility of professional expertise being replaced by technology or technology being designed for management rather than professionals [63]. There may be difficulties in harmonizing different interpretations of the technology within the organization [64]. Professionals are worried about potentially having less time with patients [44], compromising core values [63] and experiencing general fear [60]. The reluctance of personnel to adopt new systems [52] and resistance to change [54] are barriers that may be related to a lack of incentive systems [49]. Poor job satisfaction [44] and experienced stress among personnel [60] can restrain the change process and may trigger a wave of resignations due to the reform or stress it brings [66].

Factors restraining change also include phenomena related to the direction of the organization concerning the transformation. There may be active resistance to change within the organization, with a lack of belief in improvements as well as concerns about successful implementation [59]. In addition, there may be covert resistance [67] to change. If there is a sense of being an outsider in the organization concerning the development and planning of ISs [44], the reform is likely to face opposition. The organization’s inadequate learning capacity [49], especially the lack of administrative learning [46], restrain the change process. Factors restraining the direction of the reform within the organization may involve viewing technology as a limiting factor [63] or simply a lack of information [57]. External factors include a heavy reliance on partners or their lack of commitment to common changes, both of which can be obstructive factors [54].

As its worst, organizational culture can inherently restrain change [47]. Passive resistance and indifference embedded in its structures may surface during cycles of change [59]. The lack of a shared vision [49] and collaboration [53] are factors that inhibit change as well as a lack of trust between organizations [49]. Insufficient communication and tensions within the organization negatively impact not only the organization itself but also the implementation of change and ISs [64]. Hidden power dynamics [67], inappropriate behavior [60], and problems in social interactions [48] are restraining factors.

#### Technological Barriers

The complexity of the technology being implemented was identified as a restraining factor in 4 studies [46,50,53,54]. Another technological barrier may arise when software developers do not operate in the same environment where the software and tools are used [61]. If ISs are developed with a tool-centric rather than a user-centric approach [55], issues such as system underutilization [52], may occur. Technical problems [57], system downtime [44], and slow performance [44] can delay the stabilization of change. Technology can impose limitations [54] and cause workflow issues [48], which may lead to the adoption of undesirable practices [63]. Delays in transferring data from the old system to the new one [52] and the complete absence of data [44] do not support the wanted change.

In terms of user experience, the inflexibility of the system was highlighted in 2 studies [50,53]. Concerns related to the reform of ISs include issues regarding data protection, information security, and the doctor-patient relationship [55]. The potential narrowing of professional discretion [63] may be a concern for professionals. The lack of clinical efficiency and safety [65] affects the user experience. Negative previous experiences of professionals [54] influence their attitudes toward changes.

Cost factors related to ISs were identified in 2 studies [48,53]. In an ever-changing operational environment, costs can easily escalate [53]. The extensive need for customization [53] and the requirement for unique system adjustments [48] increase costs when using so called off-the-self-software. If the IS demands the use of specialized resources [53], this raises associated costs. High maintenance costs and inadequate support from the system provider to the organization are restraining factors [53].

#### Management Issues

Leadership must not leave strategic objectives vaguely defined [53]. A lack of communication regarding the vision [66], insufficient communication of the purpose [66], and inadequate presentation of goals [58] create significant obstacles to change. The lack of information [66] is one of the greatest challenges in transformations. Change initiatives may be deemed unsuccessful due to their scale [53]. If the upcoming implementation project is not adequately communicated to the personnel [59], the consequences may restrain the change.

The leader’s role is to be visible and committed to the change [66], as lack of responsibility [52] is unfavorable from the perspective of reform. It is also common for personnel to not be informed at all due to uncertainty [59], leading to feelings of abandonment among employees [66]. Challenges arise especially when management’s expectations for change conflict the values of the employees [66], such as with productivity expectations. In terms of collaboration, failure can occur in estimating the size of the project [53], often resulting in the project being larger than initially anticipated. Another identified barrier is the lack of close cooperation between clinical leadership and IT professionals [65].

#### Change Project Challenges

Inadequate communication about the project’s tasks [48] provides an undesirable start to a change project. If the participation efforts of various stakeholders are not considered [59], this can increase resistance to change. Involving end users appropriately in the selection of the system [53] drives the implementation. Regarding the implementation process, the lack of transparency and improper execution [59] were identified as barriers. Poor planning [53], delays [53], and the inability to manage a centralized timeline [48] are challenges related to project planning and execution. Budget constraints and overruns [53] are restraining factors. As for supplier collaboration, inadequate screening of suppliers [53] and a nontransparent and nonopen relationship with the supplier can hinder the process.

#### End User Concerns

End users experience concerns and fears about the impact of artificial intelligence, data privacy, and the replacement of humans by technology [45]. Among user characteristics, advanced age was identified as a restraining factor [56]. In terms of attitudes, user frustration [44] and general resistance to acceptance [57] are also barriers.

#### Institutional Barriers

As with driving factors, institutional barriers have been identified in only few studies, but they are included due to their significance. Legislative obstacles refer to factors such as data protection and security laws [46]. Regulatory factors [58] can restrain or delay IS reforms, especially in the public sector.

### A Narrative of the Change Process in IS Implementations

Based on the data, the procurement of ISs is one of the largest decisions a public sector organization can make [48]. These projects are complex [57] and often fail to meet their objectives [58,62,64]. The IS project represents a significant financial investment [48,53]. If the system proves impractical and offers limited value, it is considered a poor investment [63]. Although health care has seen rapid technological growth over the past 50 years, it has lagged behind other industries in its use of technology [54,58] and integrating technology into health care remains challenging [50].

Large health care organizations frequently face difficulties in adopting high-quality, modern technological solutions [43]. Although the benefits of systems such as EHRs are widely recognized, their potential is not always fully realized, often due to the implementation process [55]. Furthermore, the introduction of new technology has been shown to increase stress among professionals, potentially worsen existing issues such as personnel shortages and heavy workloads [44].

Changes often lead to uncertainty [66] and anxiety about how they will affect employees’ work [59]. The implementation of an IS requires time and resources from organization’s perspective [53], and it can disrupt routines [45]. In public sector, digital projects can easily become stalled, resulting delays of several years [46]. Leaders should have necessary expertise to carry out these projects [49].

It appears that the implementation of ISs remains an unresolved challenge for organizations [43,58], but innovations also offer an opportunity to transform organizational practices [67]. Over the years, technological advancements have facilitated the organization and provision of services [62], and they can radically transform how business is conducted [44,52]. For instance, the new generation of EHR systems can offer various forms of support related to patient care and operational planning [62].

ISs play a crucial role in meeting the demands for reform in public sector organizations and the use of technology has increased within public services [63]. Technology offers significant advantages in reducing errors, improving communication and enhancing patient and customer satisfaction [56]. The use of health technology in hospital setting has resulted in several benefits, such as improved services and reduced medication errors, thus promoting patient safety [61]. However, new customer and patient safety errors caused by technology continue to be a significant issue [61]. Nonetheless, electronic health care has a clear and growing impact on health care delivery worldwide and contributes to the efficiency of health care systems [51]. Digitally operated public services can make citizens’ lives easier, more satisfying, and safer [49].

## Discussion

### Principal Findings

The purpose of the study was to enhance the understanding of factors influencing the implementation of ISs in public sector organizations and how to prepare for such change. The main findings indicate that the implementation of ISs in public sector organizations is influenced by a range of driving and restraining forces. These forces can be categorized into six domains, which vary depending on whether the force is driving or restraining: (1) organizational practices and challenges, (2) technological factors and barriers, (3) management practices and issues, (4) change project factors and challenges, (5) end user factors and concerns as well as (6) institutional factors and barriers. The results align with previous research underscoring the persistent challenges in IS implementation within public sector, such as high failure rates, significant financial losses, and diminished trust in technology [6,11-16,43,64]. However, they also highlight the transformative potential of digital technologies, including improved efficiency, reduced errors, and enhanced user satisfaction [4,5,51,56,62].

Key driving forces (leadership, stakeholder involvement, and usability) and restraining forces (user resistance, lack of managerial expertise, and technical challenges) were identified as major factors influencing IS implementation public sector. These findings align with Lewin’s change theory, which emphasizes that successful IS implementation depends on overcoming restraining forces and reinforcing driving forces to facilitate a sustainable transformation [34]. The Lewin’s change model provides framework for understanding and managing the dynamics of the change as it helps to identify and analyze both driving and restraining forces to enable targeted actions to support the wanted outcome. Force field analysis helps organizations to plan their transformation journey [34,35].

Leadership support was identified as a critical driver, reducing resistance, and aligning organizational goals [43,44,49]. Leadership activities, such as addressing concerns [55,60] and fostering a shared vision for change [59] were pivotal in building trust and guiding the implementation process [64]. These findings align with earlier research highlighting the central role of active and engaged leadership in complex organizational transformations [11,18,20-22,31].

Training and stakeholder involvement were equally important, with 8 studies emphasizing pre- and postimplementation training as critical for building user competence and confidence [44,51-57]. Stakeholder participation, particularly from frontline professionals, facilitated a sense of ownership and reduced resistance, as noted in 6 studies [43,52,55,58-60].

On the other hand, user resistance [16,28-30], end users’ key restraining force, was often rooted in fears of increased administrative burdens and reduced professional autonomy [44,60]. Technical challenges, such as bad user experience [50,53,54,65], system downtime [44] and slow performance [50] further interfered with the process. Organizational silos and a lack of a shared vision were also highlighted as significant barriers to successful implementation [49,64].

Previous research emphasizes the need to understand that even the best IS alone cannot meet the expectations placed on it and it is crucial to change practices at individual and organizational level [18,21,23]. Results of this study indicates that the most significant feature of an IS is its alignment with organizational workflows [50,55,57,62], which means that the objectives set for the IS project must consider the processes and the context of the operating environment [25,26].

A narrative analysis of the introduction sections of the studies included in this scoping review revealed how previous research has described IS implementations in public sector organizations. The findings indicate that IS implementation remains a complex and unresolved challenge [43,57,58]. These projects require significant financial investment [48,53], yet they often fail to meet their objectives [58,62,64]. One major barrier is the disruption IS implementation causes to organizational routines, increasing stress among personnel and worsening workforce shortages [44,45]. Uncertainty about changes and a lack of leadership further hinder the progress [49]. Despite these challenges, the positive narrative from analyzed data emphasizes the benefits of the technology advancement and digitalization such as improved service organization [44,52,62] and increased efficiency [51] as well as reduced errors and enhanced satisfaction [49,55,61]. To conclude, studies acknowledge the significant challenges and risks involved in IS implementation, but they also emphasize the transformative potential in improving operations and service quality. This approach suggests that IS implementation is not inherently problematic, but rather that its success depends on how effectively the implementation process is managed and aligned with organizational needs.

### Limitations

This scoping review aimed to provide a broad overview and summarize the breadth and depth of the known forces influencing IS implementation in public sector organizations and to examine the narrative presented in previous search. Existing knowledge was identified, evaluated, interpreted, and synthesized. The objective of this study was not to assess the quality of the evidence, although only peer-reviewed scientific papers were included in the review. While specific search queries and inclusion criteria were applied, it is possible that some relevant studies were not captured in the process.

### Conclusions

The decision to implement an IS is a significant and complex process, often encountering challenges in achieving its objectives. These challenges relate to high costs, technical difficulties and the complexity of implementation. However, the adoption of technology also brings positive impacts. Successfully implemented ISs improve services, reduce errors, and increase customer and patient satisfaction. Despite the challenges, a well-managed implementation process can bring significant benefits for customers, organizations, and society.

This study contributes to a growing body of knowledge of public sector technology implementation, offering valuable insights for future projects, which should be examined and planned comprehensively from various identified perspectives. Previous research has primarily focused on the failure of IS implementations, highlighting the need for further studies on the factors influencing successful implementation in the public sector organizations.

## Supplementary material

10.2196/71575 Multimedia Appendix 1 Data of the review.

10.2196/71575 Multimedia Appendix 2 Narrative about information system implementations.

10.2196/71575 Checklist 1 PRISMA-ScR (Preferred Reporting Items for Systematic reviews and Meta-Analyses extension for Scoping Reviews) checklist.

## Abbreviations

EHR: electronic health record
IS: information system
IT: information technology
PRISMA: Preferred Reporting Items for Systematic Reviews and Meta-Analyses
PRISMA-ScR: Preferred Reporting Items for Systematic Reviews and Meta-Analyses Extension for Scoping Reviews
