# Supplementary material for: Driving and Restraining Forces in the Implementation of Information Systems in the Public Sector: Scoping Review
Source: JMIR Hum Factors. 2025 Jun 11;12:e71575. doi: 10.2196/71575 (PMC12176313; doi:10.2196/71575)
Supplement: Multimedia Appendix 1 [file humanfactors-v12-e71575-s001.pdf]

Multimedia Appendix 1. Data of the review

No.	Author, Year, Title, Country	The purpose of the study	Method and data	Driving factors	Restraining factors	Public sector domain
1	Shimange et al., 2023<br><br>A South African institution perspective of a framework for enterprise resource planning systems<br><br>South Africa	To investigate the potential for South African government to implement guidelines that would support the implementation of a sustainable Enterprise Resource Planning (ERP) system, with aim of improving their information system strategy.	A mixed-methods study: A survey (n = 20) in which quantitative data was collected through closed-ended questions and qualitative data through open-ended questions.	**Information system:**<br>- Cost and ease of maintenance<br>- Systems meets organizational requirements<br>- Fits organizational culture<br>- Passes quality gates<br><br>**Change project:**<br>- Proper project planning<br>- Effective change management practices<br><br>**Management:**<br>- Support from top management<br><br>**Organizational:**<br>- Specialized expertise of end users<br>- End-user training before and after implementation<br>- Readiness for change<br>- Availability of internal technical resources	**Information system:**<br>- System complexity for the end user<br>- The system requires extensive customization and expert resources for task execution, leading to implementation delays and increased costs in an ever-changing operational environment<br>- Lack of system flexibility, causing processes to be adapted to fit the system<br>- Maintenance costs<br>- Inadequate support from the system vendor<br><br>**Change project:**<br>- Budget<br>- Inadequate screening of vendors<br>- Implementation delays and cost overruns<br>- Poorly planned project<br>- End users not involved in system selection	Taxation

					**Management:**<br>- Lack of clear formulation of strategic objectives<br>- Project size not considered during the planning phase<br><br>**Organizational:**<br>- Organizational structure<br>- Lack of collaboration within the organization	
2	Kiepek et al., 2019<br><br>An Evaluation of System End-User Support during Implementation of an Electronic Health Record Using the Model for Improvement Framework<br><br>United States of America	To evaluate the initial support processes for end users during the implementation of a patient information system at a university hospital, and to identify the factors that enable success, challenges, and lessons learned.	A qualitative case study. Utilizing the continuous improvement PDSA model, the study describes activities that follow the concepts of Planning, Doing, Studying, and Acting, in providing support to end users during the implementation of an electronic patient information system.	**Change project:**<br>- Clear roles<br>- Strong communication practices<br>- Command center during implementation<br><br>**Management:**<br>- Participation<br>- Support during planning and execution<br><br>**Organizational:**<br>- Ability to make decisions swiftly and timely	**Information system:**<br>- Unique system customizations reduce the level of external resource knowledge<br>- Workflow issues<br><br>**Change project:**<br>- Lack of open and transparent relationship with the vendor<br>- Mismanagement and neglect of centralized scheduling<br>- Tasks of the project are not communicated to support staff<br>- Logistics for external personnel	Healthcare

					(accommodation and transportation)<br><br>**Organizational:**<br>- Organizational culture<br>- Social interactions	
3	Nitiéma, 2023<br><br>Artificial Intelligence in Medicine: Text Mining of Health Care Workers´ Opinions<br><br>United States of America	To investigate healthcare workers' perceptions of adopting and implementing artificial intelligence technology in the healthcare sector.	Qualitative study using structural thematic modeling. The data consisted of comments on artificial intelligence from 905 healthcare professionals published on an online forum (from at least 77 countries) between May 2013 and October 2021. Structural thematic modeling was used to identify discussion topics, and hierarchical clustering was performed to determine how these topics grouped into different categories.	**Information system:**<br>- Address concerns through technological solutions.<br>- Potential to improve the quality of care.<br><br>**Change project:**<br>- Involvement of stakeholders.<br><br>**Organizational:**<br>- Address concerns with authorities, including agencies responsible for the safety of healthcare products.<br>- Clearly define the role of artificial intelligence in care functions and obtain feedback and comments from employees to adjust the AI implementation plan to the organization.<br>- Support for employees is essential for successful technology adoption.	**Organizational:**<br>- Concerns that new technology (AI) might replace human jobs.<br>- Adoption of AI to improve care quality and organizational outcomes may increase stress among healthcare professionals.<br>- Worries that technology could diminish the importance of physical examination and the value of human touch and empathy.<br>- Concerns about the deterioration of the patient-clinician relationship and legal liability when using AI.<br>- Worries related to health equity and access to care.<br>- Concerns about the reliability and accuracy of	Healthcare

				- Organizational support.<br>- Ethical guidelines regarding the role of AI in patient-clinician relationships.	information produced by AI applications. Skepticism about AI's accuracy can undermine trust in AI applications and complicate their adoption and use.<br>- Privacy concerns.	
4	Morrison et al., 2022<br><br>Between a rock and a hard place: Nurse managers' experiences of large-scale organizational change in the public health service<br><br>Denmark	To investigate nurse managers´ experiences with major organizational changes and their impact on their work life and working conditions.	Qualitative phenomenological-hermeneutic research using Ricœur's analytical method. Semi-structured interviews with nurse managers (n = 12) at a public sector university hospital. A three-phase analysis process is employed to aid in interpretation and understanding, deepening the significance of the research from the nurse managers´ perspectives.	**Change project:**<br>- Support structures embedded within the change process.<br>- Employee involvement in the change process.<br><br>**Organizational:**<br>- A guiding vision for the direction of the change.<br>- Peer support.<br>- Employee determination to overcome the change.<br>- Support at all levels, including those in leadership roles.	**Management:**<br>- Employees left to fend for themselves.<br>- Absence of visible and committed leadership.<br>- Lack of information.<br>- Inadequate communication of the vision.<br>- Poor communication of the purpose of the change.<br>- Conflict between management expectations and employee values (e.g., quality care versus efficiency demands).<br><br>**Organizational:**<br>- Resignations due to the change or associated stress.	Healthcare

5	Hörhammer et al., 2021<br><br>Building Primary Health Care Personnel's Support for a Patient Portal While Alleviating eHealth-Related Stress: Survey Study<br><br>Finland	To investigate the impact of management implementation practices, the usability of information technology (IT), and staff eHealth competencies on the adoption of patient portals and the support of eHealth-related stress among healthcare personnel.	Data was collected via a survey (n = 919) from two Finnish organizations. Linear and logistic regression analyses were employed to examine the relationships between variables.	**Information system:**<br>- Good usability.<br>- Adequate support.<br><br>**Organizational:**<br>- Effective implementation practices.<br>- Staff eHealth competency.	-	Healthcare
6	Sarwar et al., 2019<br><br>Children's services in the age of information technology: What matters most to frontline professionals<br><br>United Kingdom	To investigate how technological changes impact public administration and service delivery.<br><br>To gain a better understanding of the technology adoption process in public organizations and to inform the development of technology that supports practitioners	Case Study (n=3): The cases were selected based on the number of residents and the composition of service provision. Data were collected through semi-structured interviews conducted with professionals at various hierarchical levels within each case study site. The interview data were analyzed using a three-phase coding process.	**Information system:**<br>- Assists in the execution of professional tasks.<br>- System development aligns with the functional and professional tendencies of users.<br><br>**Organizational:**<br>- Changes are in harmony with the professionals' own values.<br><br>**Management:**<br>- Decision-makers and users perceive and interpret the	**Information system:**<br>- Narrowing of discretion.<br>- Use of non-recommended practices.<br>- Tool-driven rather than user-driven.<br><br>**Organizational:**<br>- New technology implemented without considering professionals, only management.<br>- Professional efficiency replaced by performance evaluation techniques.	Social welfare

		in their daily professional work.		technology in the same way, share the same objectives, and interact with it in a cohesive manner.	- Inadequate implementation due to the perception of technology as restrictive, leading to apparent resistance.<br>- Compromise on core values.<br>- Professional expertise is not replaced by technology.	
7	Bernardi et al., 2019<br><br>Clinical managers' identity at the crossroad of multiple institutional logics in it innovation: The case study of a health care organization in England<br><br>United Kingdom	The aim is to investigate how clinical leaders utilize their hybrid identities to reconcile differences between competing institutional logics that influence IT innovation. This exploration is particularly relevant as not all clinical leaders are either capable of or willing to support IT innovations, especially when these innovations challenge their established professional practices.	A case study approach was employed, focusing on three examples of IT innovations: digitally enabled cardiac imaging and two telehealth projects. Data were collected through semi-structured interviews (n = 18) and observation.	**Management:**<br>- Clinical leaders promote the acceptance of IT innovations.<br>- Leaders negotiate tensions between professional boundaries and institutional logics.<br>- Demonstrating trust to build bridges.<br>- The hybrid role of leaders is also common in digital transformations beyond healthcare.<br><br>**Organizational:**<br>- Users exert effort to reconcile professional norms with IT innovation standards related to standardization and efficiency.<br>- Professional values and	**Information system:**<br>- Lack of clinical effectiveness.<br>- Lack of clinical safety.<br><br>**Management:**<br>- Clinical leaders acting solely as representatives of medicine without close collaboration with IT professionals and other professional groups.	Healthcare

				cultures are incorporated to support digital transformation.		
8	Bunduchi et al., 2019<br><br>Coping with institutional complexity. Intersecting logics and dissonant visions in a nation-wide healthcare IT implementation project<br><br>United Kingdom	To investigate how digital transformation projects involving the large-scale implementation of new information technology progress institutionally within complex organizations, where stakeholders must reconcile multiple demands and expectations while developing diverse perspectives on technology.	A qualitative case study of a public sector technology project was conducted. Data collection involved semi-structured interviews (n=25), including 19 individual interviews and 6 group interviews. The interviews were triangulated with document analysis to provide a comprehensive view of user experiences and the broader context of the project. The analysis began with open coding of transcriptions.	**Organizational:**<br>- Consistency of internal perspectives within groups and between them.<br>- Strategic action to shape the project versus passive action as a follower.<br>- The role of the "organizational vision" (the collective interpretation of the new technology being implemented in their organization) in stakeholders' responses and how these responses can influence IT implementation project processes and outcomes in complex institutional environments.<br>- Understanding how complexity can shape stakeholders' responses.<br><br>**Management:**<br>- Ensuring that the innovation's guiding vision is	**Organizational:**<br>- Tensions between multiple logics.<br>- Difficulties in aligning diverse and evolving interpretations of technology among different stakeholder groups.<br>- Interaction.<br>- Complexity of modern organizations.	Healthcare

				widely understood, consistent, and stable over time.<br>- Reconciling perspectives before conflicting views become entrenched.		
9	Kemal et al., 2023<br><br>Digital Innovation in Social Cash Organizations - the Effects of Institutional Interactions for Transforming Organizational Practices<br><br>Pakistan	Examine, based on a neo-institutional perspective, the unique institutional interactions influencing the transition to digital social cash payments and the creation of new institutional arrangements within social cash organizations.	Case study. Qualitative data were collected through thirty (n=30) semi-structured interviews with key personnel and stakeholders within the organization.	**Institutional:**<br>- Technological and economic.<br>- Regulatory.<br>- Social and organizational.<br>- International.<br>- Political.<br><br>**Information system:**<br>- Institutionalization of technology.<br>- Termination of old practices.<br><br>**Organizational:**<br>- Openness/visibility<br>- Efficiency/safety	**Organizational:**<br>- Covert power<br>- Resistance of hidden forces	Social welfare

10	Agyei-Ababio et al., 2023<br><br>Digitalization of revenue mobilization in an emerging economy: the new institutional theory perspective<br><br>Ghana	Investigate how the institutional environment, composed of regulatory, normative, and cognitive factors, affects technology use in the public sector. Specifically, examine how institutional factors influence the adoption of information technology for revenue mobilization within the tax administration of an emerging economy.	Qualitative case study. Data were collected through semi-structured interviews (n=11), informal discussions, and observations. Themes were identified and information was organized accordingly.	**Organizational:**<br>- Values<br>- End-user training<br>- Organizational support<br>- Inclusion of all stakeholders<br>- Obligation to use the system<br><br>**Institutional:**<br>- Governmental regulation<br><br>**Management:**<br>- Change management<br><br>**Change project:**<br>- Establishment of a project team	**Organizational:**<br>- Reluctance to adopt the new system<br>- Acceptance of the new system<br><br>**Information system:**<br>- Underutilization of the system<br>- Delays in data migration<br><br>**Management:**<br>- Inadequate responsibility and ownership of the system	Taxation
11	Benedicts et al., 2020<br><br>Electronic Medical Records implementation in hospital: An empirical investigation of individual and organizational determinants	Integrate institutional and individual factors to explain which elements may trigger or hinder the adoption of patient information systems in hospitals and which variables leaders can leverage to influence professional behavior.	Qualitative exploratory survey research with 15 questions directed at physicians and nurses (n = 114), case study.	**Information system:**<br>- Perceived usefulness<br>- Perceived ease of use<br><br>**Organizational:**<br>- Influence of leading peer groups (innovation masters)<br>- Time required to regain control over tasks and processes by professionals<br>- Immediate inclusion of frontline professionals	**Institutional:**<br>- Regulatory factors<br><br>**Management:**<br>- Inadequate presentation of objectives by leadership	Healthcare

	Italy			- Understanding of the foundations of technological change<br><br>**Management:**<br>- Change management<br>- User motivation<br>- Context-specific objectives		
12	Han et al., 2020<br><br>Factors Influencing the Adoption of Health Information Standards in Health Care Organizations: A Systematic Review Based on Best Fit Framework Synthesis<br><br>China	The goal is to develop a comprehensive framework of factors that influence the acceptance and adoption of health information standards within healthcare organizations.	A systematic literature review was conducted, including 35 articles. A synthesized, most applicable framework was then developed, incorporating thematic analysis of the included articles.	**Information system:**<br>- Relative value<br>- Compatibility<br>- Trialability<br>- Observability<br>- External pressure<br>- External support<br><br>**Organizational:**<br>- Organizational culture<br>- Staff training<br>- Readiness<br>- Trust in partners<br><br>**Management:**<br>- Support from top management	**Information system:**<br>- Complexity<br>- Previous experiences<br>- Technology-related limitations<br><br>**Organizational:**<br>- Resistance to change<br>- Dependence on partners<br>- Commitment of partners	Healthcare

13	Kayser et al., 2022<br><br>Health Professionals' eHealth Literacy and System Experience Before and 3 Months After the Implementation of an Electronic Health Record System: Longitudinal Study<br><br>Denmark	To investigate how a recently developed and modified instrument for measuring staff electronic health literacy can be utilized to inform both the system provider and the healthcare organization during the adoption process, and to assess whether staff perceptions of ease of use change, as well as how this may relate to the level of electronic health literacy among healthcare staff.	A qualitative study was conducted. A modified version of the eHLQ (Electronic Health Literacy Questionnaire) was distributed to the staff (n = 295) before the implementation of a new electronic patient record system and three months thereafter.	**Information system:**<br>- Positive user experience<br>- Ease of use<br>- Customizability of the user interface<br>- Presentation of system functions according to users' specific needs and expertise<br>- Users' technology proficiency<br>- Meets needs<br>- Supports workflows<br>- Information availability<br><br>**Organizational:**<br>- Identification of users who require special support and training	-	Healthcare
14	Stefánsdóttir et al., 2022<br><br>Implementing a new emergency department: a qualitative study of health professionals' change responses	To examine how leaders and key personnel respond to the planned change in the emergency department and how they perceive the change associated with the implementation of the new department.	A qualitative study involving semi-structured individual interviews (n = 51). The interviews were transcribed, and responses categorized as reactions to the change were systematically placed into a deductive coding framework (Excel).	**Organizational:**<br>- Commitment<br>- Professional moral and ethical obligation to promote change<br>- Involvement of professionals<br>- Support for change<br>- Belief in success<br><br>**Management:**<br>- Communication	**Organizational:**<br>- Indifference<br>- Concerns about success<br>- Passive resistance<br>- Attempts to participate are ignored<br>- Active resistance<br>- Lack of transparency in the adoption process<br>- Adoption process inadequately implemented	Healthcare

	Denmark			- Leaders' ability to address staff questions about the change<br>- Listening to employees who resist the change to increase mutual understanding	- Lack of belief in improvements<br><br>**Management:**<br>- Adoption process not communicated<br>- Staff not informed due to uncertainty	
15	Pittaway et al., 2020<br><br>Know-how to lead digital transformation: The case local governments<br><br>Canada	To investigate the knowledge and skills that leaders need to manage digital transformation.	Qualitative case study (n = 11) of established local government actors in Canada. Semi-structured interviews.	**Management:**<br>- Expertise<br>- Authority of the IT manager<br>- Leadership-driven, collaborative IT team<br>- Monitoring performance deficiencies<br><br>**Organizational:**<br>- Analytical organizational strategy aligned with IT strategy<br>- Building around integrated organizational processes<br>- Reforming IT governance<br>- Shared innovation vision	**Organizational:**<br>- Inadequate learning capability<br>- Lack of a shared vision<br>- Absence of inter-organizational trust<br>- Lack of incentive systems	Government

16	Giulio et al., 2018<br><br>Multilevel policy implementation and the where learning: the case of the information system for school buildings in Italy<br><br>Italy	To explain three distinct phases related to adoption: 1) failure of the national program, 2) success of local solutions, and 3) revision of the national adoption strategies.	Qualitative longitudinal case study involving interviews with local government leaders and registry officials (n = 6).	**Organizational:**<br>- Adequate financial resources<br>- Strategy<br>- Centralized coordination and standardization functions<br>- Allow market actors to develop effective solutions<br>- Learning through negotiation and social interaction<br><br>**Information system:**<br>- Feasibility<br>- Innovative solutions are likely to emerge through local development<br>- Easy scalability	**Information system:**<br>- Excessive complexity<br><br>**Institutional:**<br>- Legislative barriers (security legislation, data protection)<br><br>**Organizational:**<br>- Lack of administrative learning	Government
17	Haverinen et al., 2022<br><br>National Development and Regional Differences in eHealth Maturity in Finnish Public Health Care: Survey Study	The goal is to investigate the national development of electronic healthcare maturity levels in primary and specialized healthcare in Finland from 2011 to 2020.<br><br>The second goal is to examine regional differences in	Qualitative study. Data were collected over four different years using an online survey from the ICT Use in Finnish Healthcare project. The state of electronic healthcare was described by 16 indicators. Participants included those from specialized healthcare (n = 21) and	**Institutional:**<br>- Legislative changes<br>- National strategies<br><br>**Information system:**<br>- Smart devices<br>- Telecommunication networks<br>- Technical support<br><br>**Organizational:**<br>- Advanced maturity level	-	Healthcare

	Finland	electronic healthcare maturity levels among Finnish hospital districts in 2020.	primary healthcare (n = 121–139).	- Use of a consistent electronic patient record system brand<br>- Early investment in development<br>- Dedicated internal work<br>- Staff training		
18	Jedwab et al., 2022<br><br>Nurses' Experiences After Implementation of an Organization-Wide Electronic Medical Record: Qualitative Descriptive Study<br><br>Australia	The goal is to investigate Australian nurses' experiences with EMR (Electronic Medical Record) systems after organizational-level EMR implementation, to develop future technology adoption strategies that enhance nurses' work, workflows, and well-being.	Qualitative descriptive study. Focus group and individual interviews with nurses (n = 158) and surveys with open-ended questions conducted across six hospital sites. The analysis included complementary inductive and deductive approaches. Reflexive thematic analysis followed the use of a theoretical domain framework for coding barriers or facilitators to EMR (Electronic Medical Record) usage.	**Organizational:**<br>- User motivation<br>- Encouragement<br>- User training<br>- User commitment<br>- Safe space for expressing concerns<br><br>**Management:**<br>- Change management	**Organizational:**<br>- Reduced time with patients<br>- Feeling excluded from development and design<br>- Concerns about patient safety<br>- Concerns about workforce retention<br>- Concerns about losing professional identity<br>- Concerns about decreased visibility of work<br>- Poor job satisfaction<br>- Competing work demands<br><br>**Information system:**<br>- Missing information<br>- Slow usability<br>- System outages<br>- User frustration	Healthcare

19	Raza et al., 2023<br><br>Organizational Change and Workplace Incivility: Mediated by Stress, Moderated by Emotional Exhaustion<br><br>Pakistan	To investigate the impact of emotional exhaustion on the relationship between stress and workplace incivility in the public sector.	Quantitative study. The relationship between organizational change and workplace incivility was examined through the lens of resource conservation theory. Hypotheses were tested using a time-lagged approach with data collected from (n = 262). Data on the independent variable and mediator were collected at T1, while data on the moderator and dependent variable were collected at T2, with a one-month interval between the two periods.	**Organizational:**<br>- Employee effort<br>- Employee commitment<br>- Involvement of employees in the change process<br>- Employees as part of the change<br><br>**Management:**<br>- Management must ensure the successful implementation of the change process<br>- Communicate the goals of the change to employees to avoid incivility<br>- Management accepts employees' suggestions and concerns about the change<br>- Necessary actions to address any ambiguities or issues	**Organizational:**<br>- Uncertainty<br>- Fear<br>- Insecurity<br>- Stress<br>- Cycle of changes<br>- Incivility	Government

20	Ndabu et al., 2021<br><br>Perceptual Gaps Between Clinicians and Technologists on Health Information Technology-Related Errors in Hospitals: Observational Study<br><br>United States of America	The aim is to categorize technology-related medical errors in the hospital environment using an extended version of the sociotechnical framework, in order to understand significant differences in the perspectives of clinical and technology stakeholders regarding the possible causes of these errors.	Qualitative study. Medical errors (n = 67) were collected from previous research (n = 36), identified from leading healthcare databases. Errors were selected from the primary list that occurred in hospital environments. Semi-structured interviews were conducted with five medical professionals and six IT professionals to map the events across various dimensions of the extended sociotechnical framework.	**Information system:**<br>- Adequate testing practices<br>- Adequate validation practices<br>- Software developers temporarily placed in clinical environments to enhance understanding<br>- Software developers sensitive to provider and patient needs<br>- Good user experience<br>- Good software design<br>- Implementation strategy	**Information system:**<br>- Software-specialized experts do not operate in the environment where the software and tools are actually used.	Healthcare

21	Fennelly et al., 2020<br><br>Successfully implementing a national electronic health record: a rapid umbrella review<br><br>Ireland	To identify and examine the key factors that influence the success of electronic patient record system implementation across different healthcare contexts	Umbrella literature review (n = 27) and expert panel (n = 10).	**Change project:**<br>- Implementation project management<br>- Executives (CIOs)<br>- Project management team<br>- Good, confidential relationships with vendors and consulting firms<br>- Clear implementation strategy<br>- Goals (measurable and clear)<br>- Implementation process<br>- Clear roles and division of labor<br>- Resource allocation<br><br>**Management:**<br>- Leadership<br>- Independent from top management<br>- Listening to concerns<br><br>**Organizational:**<br>- Organizational culture<br>- Engagement<br>- Internal and external communication<br>- User training<br>- IT skills<br><br>**Information system:**	**Information system:**<br>- Concerns about data privacy<br>- Concerns about data security<br>- Concerns about the patient-doctor relationship<br><br>**Organizational:**<br>- Concerns about roles<br>- Concerns about responsibilities	Healthcare

				- Usability<br>- Interoperability<br>- Technical support<br>- Workflows<br>- Perceived usefulness<br>- IT infrastructure and tools<br>- Adaptability<br>- Testing<br><br>**Institutional:**<br>- National standards		

22	Tossaint-Schienmakers et al., 2021<br><br>The Challenge if Integrating eHealth Into Health Care: Systematic Literature Review of the Donabedian Model of Structure, Process, and Outcome<br><br>Holland	To investigate whether indicators related to the successful integration of electronic healthcare into healthcare can be identified within structure, process, and outcome categories, and to examine which structure and process indicators are associated with outcome indicators.	Using Donabedian's Structure-Process-Outcome (SPO) framework, a systematic literature review (n = 11) was conducted to identify indicators related to the integration of electronic healthcare into healthcare organizations. Data extraction forms provided an overview of the study characteristics, electronic health features, and indicators. Extracted indicators were categorized into themes and sub-themes within the structure, process, and outcome categories	**Information system:**<br>- Technology must be well-aligned with the organization's structure and daily care processes<br>- Adaptability to the local context<br>- Usability<br>- Integration into clinical workflows<br><br>**Organizational:**<br>- Adapting processes<br>- Utilization of human resources<br>- Monitoring of processes<br>- Communication<br><br>**Management:**<br>- Communicating expectations<br>- Staff requirements<br>- Processes	**Information system:**<br>- Rigidity<br>- Complexity	Healthcare
23	Esdar et al., 2021<br><br>The Effect of Innovation Capabilities of Health Care Organizations on the Quality	The aim is to explain how key constructs of organizational innovation capabilities relate to the quality of health technology, based on a conceptual sociotechnical model of innovation and	A survey was conducted to evaluate various aspects of health technologies, organizational innovation capabilities, and contextual variables. The survey was administered to Chief Information	**Organizational:**<br>- Innovation-friendly attitude<br>- User engagement<br>- Openness<br>- Ability to create space for creativity, agility, and communication<br>- Professional information management	-	Healthcare

	of Health Information Technology: Model Development With Cross-sectional Data<br><br>Germany	quality known as the IQHIT model, and to help define how information access could be improved in healthcare organizations.	Officers in hospitals in Austria, Germany, and Switzerland. Data from 232 hospitals were used for empirical validation of the model using partial least squares structural equation modeling to identify associations as well as mediating and moderating effects.	- Positive attitude towards information technology<br><br>**Change project:**<br>- Implementation practices<br><br>**Management:**<br>- Crucial role of top management<br>- Communication<br>- Leadership practices		
24	Al-Otaibi et al., 2022<br><br>The Factors Contributing to Physicians' Current Use of and Satisfaction With Electronic Health Records in Kuwait's Public Health Care: Cross-sectional Questionnaire Study<br><br>Kuwait	To investigate factors related to the implementation of patient information systems and the proliferation of these systems in Kuwait, and to identify predictors of physician satisfaction with patient information systems in public hospitals in Kuwait.	Quantitative study. Primary data were collected using questionnaires distributed to 295 physicians. The data were analyzed in SPSS using linear regression, adjusted for demographic variables.	**End users:**<br>- Female gender<br>- High education level<br><br>**Information system:**<br>- Perceived usefulness<br>- Duration of use<br>- Prediction of positive effects<br>- Ease of use<br><br>**Organizational:**<br>- Making benefits visible<br>- User training	**End users:**<br>- Advanced age	Healthcare

25	Saiyed et al., 2022<br><br>The Rapid Implementation of an Innovative Virtual Diabetes Boot Camp Program: Case Study<br><br>United States of America	The goal is to develop a virtual boot camp program for patients with diabetes within 3 months, from project planning to the registration of the first patients. The objective is to provide practical strategies for the rapid launch of an effective virtual program to improve diabetes management.	Qualitative study. Physicians, dietitians, and trainers formed a multidisciplinary team to develop a virtual program for patients with diabetes, with support from a telemedicine team. The program combined online diabetes data monitoring with weekly telemedicine visits for a duration of 12 weeks.	**Information system:**<br>- Integrated workflows<br><br>**Organizational:**<br>- User training<br>- Strategy<br>- Ongoing effort<br>- Commitment<br><br>**Management:**<br>- Stakeholder group composed of leaders	**End users:**<br>- Lack of acceptance<br><br>**Information system:**<br>- Technical issues<br><br>**Organizational:**<br>- Lack of information	Healthcare
