# Supplementary material for: Driving and Restraining Forces in the Implementation of Information Systems in the Public Sector: Scoping Review
Source: JMIR Hum Factors. 2025 Jun 11;12:e71575. doi: 10.2196/71575 (PMC12176313; doi:10.2196/71575)
Supplement: Multimedia Appendix 2 [file humanfactors-v12-e71575-s002.pdf]

Multimedia Appendix 2. Narrative about IS implementations

No	Author, Year	What kind of narrative is told about implementations?	Grouping
2	Kiepek et al. 2019	One of the biggest decisions	Procurement
7	Bernardi et al., 2019	Primarily complex projects	Procurement
8	Bunduchi et al., 2022	Frequently fail to meet their objective	Procurement
11	Benedicts et al., 2024	Successful IT projects remain rare	Procurement
1	Shimange et al., 2023	High costs	Investment
2	Kiepek et al. 2019	A significant financial investment	Investment
6	Sarwar et al., 2022	The systems are impractical, offer limited value to professionals, hence poor investment	Investment
1	Shimange et al., 2023	Consumes resources	Implementation
1	Shimange et al., 2023	Consumes time	Implementation
3	Nitiéma, 2023	Can disrupt routines	Implementation
5	Hörhammer et al., 2021	Success depends on usage	Implementation
5	Hörhammer et al., 2021	Effective implementation practices facilitate the adoption of use	Implementation
5	Hörhammer et al., 2021	Efficient user support	Implementation
13	Kayser ym., 2024	Not every implementation has succeeded	Implementation
18	Jedwab ym., 2023	The implementation of new healthcare technology has been shown to increase nurses' stress; therefore, such implementation may exacerbate existing issues, such as nurse shortages and high workloads.	Implementation
21	Fennelly ym., 2020	Although the widely recognized benefits of electronic patient record systems are known, their full potential is not always realized, often as a result of the implementation process.	Implementation
23	Esdar ym., 2021	Larger healthcare organizations often struggle to implement high-quality and modern technology solutions.	Implementation
1	Shimange et al., 2023	A substantial shift towards a positive outcome	Change
2	Kiepek ym., 2021	Major impact on change	Change
4	Morrison ym., 2022	Creates a sense of uncertainty among staff	Change
4	Morrison ym., 2022	Leaders' stress can be transmitted to their subordinates	Change

5	Hörhammer et al., 2021	Engagement with digital tools can easily fail	Change
8	Bunduchi et al., 2022	Complex	Change
8	Bunduchi et al., 2022	Long-lasting	Change
8	Bunduchi et al., 2022	Challenging to execute	Change
10	Agyei-Ababio et al., 2023	Technology development is drastically altering the manner in which business is carried ou	Change
11	Benedicts et al., 2020	Healthcare professionals oppose patient record systems	Change
13	Kayser et al., 2022	Over the past 50 years, technological and digital advancements have facilitated the transformation of healthcare organization and delivery.	Change
14	Stefánsdóttir et al., 2022	Changes create uncertainty and anxiety about how the change will affect employees' work.	Change
15	Pittaway et al., 2020	Digital transformation has stalled because leaders lack the necessary expertise to replace old systems with new ones.	Change
16	Giulio et al., 2018	Public sector digitalization projects can easily get stuck, leading to implementation delays of several years.	Change
17	Haverinen et al., 2022	However, the use of information and communication technology in healthcare requires strategic and comprehensive national actions to be utilized in the most effective way.	Change
18	Jedwab et al., 2022	The implementation of patient information systems causes significant changes in nurses' workplaces, work, and workflows.	Change
19	Raza et al., 2023	One of the main reasons for the failure of organizational change focuses on technical aspects, such as information systems.	Change
8	Bunduchi et al., 2022	Many expectations and values within the organization	Organization
8	Bunduchi et al., 2022	Different interpretations of technologies	Organization
9	Kemal et al., 2023	Innovations provide an opportunity to transform organizational practices.	Organization
11	Benedicts et al., 2020	The system implementation remains an unresolved challenge	Organization
11	Benedicts et al., 2020	Healthcare has lagged behind other sectors in the use of technology.	Organization
22	Tossaint-Schienmakers et al., 2021	Integrating electronic health care into healthcare systems is, however, challenging.	Organization
23	Esdar et al., 2021	Large healthcare organizations struggle with building complex health information technology solutions and face ever-increasing pressure to continuously innovate their information systems.	Organization

5	Hörhammer et al., 2021	Good usability supports the adoption of use.	Technology
6	Sarwar et al., 2022	Usage of ICT solutions has grown in public services.	Technology
6	Sarwar et al., 2022	Systems play a significant role in requirements for modernization.	Technology
7	Bernardi et al., 2019	Different stakeholders have varying expectations of IT innovation	Technology
7	Bernardi et al., 2019	Different expectations slow down the adoption of technology.	Technology
11	Benedicts et al., 2020	Technology has the potential to support clinical practices	Technology
12	Han et al., 2020	Rapid technological growth in healthcare over the past 50 years	Technology
12	Han et al., 2020	Systems are complex	Technology
13	Kayser et al., 2022	The new generation of patient information systems can provide various forms of support for patient care as well as for operational planning.	Technology
15	Pittaway et al., 2020	Digitally enabled public services can make citizens' daily lives easier, more satisfying, and safer.	Technology
17	Haverinen et al., 2022	Electronic health care has a clear and growing impact on the delivery of healthcare worldwide and the efficiency of healthcare systems.	Technology
20	Ndabu et al., 2021	The widespread use of information technology has enhanced patient safety in hospital settings.	Technology
20	Ndabu et al., 2021	In hospital settings, the use of health technology has yielded several benefits, such as improved healthcare services and a reduction in medication errors.	Technology
20	Ndabu et al., 2021	New patient safety errors resulting from technology remain a significant problem.	Technology
24	Al-Otaibi et al., 2022	Technology offers significant benefits in healthcare through error reduction, improved communication, and enhanced patient satisfaction.	Technology
25	Saiyed et al., 2022	As telemedicine advances, there is a need to implement programs effectively that respond quickly to patients' needs.	Technology
